# Transcription Factor Control of Lymphatic Quiescence and Maturation of Lymphatic Neovessels in Development and Physiology

**DOI:** 10.3389/fphys.2021.672987

**Published:** 2021-11-02

**Authors:** Zarah B. Tabrizi, Nada S. Ahmed, Joseph L. Horder, Sarah J. Storr, Andrew V. Benest

**Affiliations:** ^1^Endothelial Quiescence Group, University of Nottingham, Nottingham, United Kingdom; ^2^Nottingham Breast Cancer Research Centre, Centre for Cancer Sciences School of Medicine, Biodiscovery Institute, University of Nottingham, Nottingham, United Kingdom

**Keywords:** lymphatic, lymphangiogenesis, transcription factor, quiescence, inflammation

## Abstract

The lymphatic system is a vascular system comprising modified lymphatic endothelial cells, lymph nodes and other lymphoid organs. The system has diverse, but critical functions in both physiology and pathology, and forms an interface between the blood vascular and immune system. It is increasingly evident that remodelling of the lymphatic system occurs alongside remodelling of the blood microvascular system, which is now considered a hallmark of most pathological conditions as well as being critical for normal development. Much attention has focussed on how the blood endothelium undergoes phenotypic switching in development and disease, resulting in over two decades of research to probe the mechanisms underlying the resulting heterogeneity. The lymphatic system has received less attention, and consequently there are fewer descriptions of functional and molecular heterogeneity, but differential transcription factor activity is likely an important control mechanism. Here we introduce and discuss significant transcription factors of relevance to coordinating cellular responses during lymphatic remodelling as the lymphatic endothelium dynamically changes from quiescence to actively remodelling.

## The Lymphatic System

The lymphatic vascular system comprises a hierarchical system of lymphatic capillaries, that drain into higher calibre collecting vessels that return protein rich lymph (generated from interstitial fluid) and trafficking cells (e.g., lymphocytes and myeloid) back into the venous circulation. As the lymphatic system evolved it allowed higher order eukaryotes to have a regulated fluid balance system. (Lymphatic vessels are found in reptiles, amphibians, birds and mammals) (Adams and Alitalo, 2007).

Extravasated water, solutes and cells are forced out the vascular system at higher pressure, resulting in fluid leakage from the permeable capillaries into the interstitium. The lymphatic system resorbs this fluid.

**FIGURE 1:**
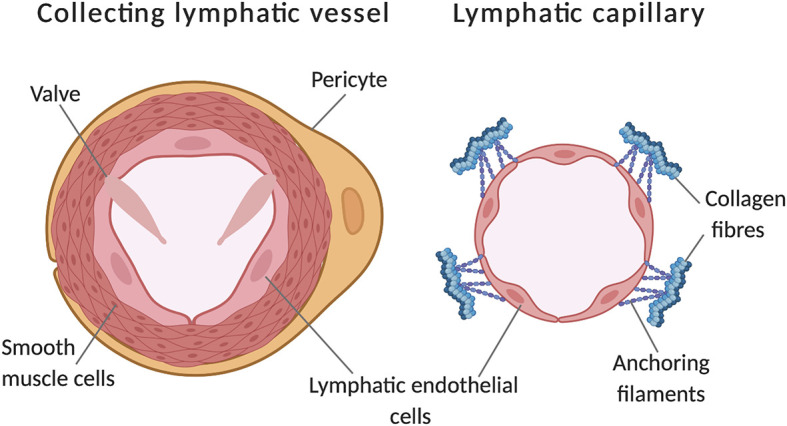
Comparison of a lymphatic collecting vessel and a lymphatic capillary. Further to lumen calibre differences, additional features of collecting vessels include pericyte coverage, a layer of smooth muscle cells and the presence of valves to prevent backflow. Capillaries have discontinuous junctions between endothelial cells, these act as sights of leukocyte entry and increase the permeability of the vessel. Additionally, capillaries are connected to the extracellular matrix by anchoring filaments, these become taut in places of swelling, opening the lumen to allow drainage of tissue fluid. Adapted from Tammela and Alitalo (2010). Made with Biorender.com.

Insufficient fluid resorption, resulting in fluid accumulation in tissue results in pathological swelling (oedema) (Dagenais et al., 2004). The interstitial fluid enters the lymphatic vessel through modified intracellular junctions (Yao et al., 2012) and is pumped through capillaries to valve containing collecting vessels (Figure 1; Escobedo and Oliver, 2017). This occurs via smooth muscle cell mediated coordinated contractions with lymph returned to the vascular network through lymphovenous valves. Lymphatic valves, (and lymphovenous valves) have been identified as containing their own subtype of lymphatic endothelial cells, a transcriptionally unique signature identified by Takeda et al. (2019) by single-cell RNA sequencing (Takeda et al., 2019).

## Lymphangiogenic Mechanisms

Lymphangiogenesis is the growth of lymphatic vessels, which occurs primarily from sprouting lymphatic vessels that arise from embryonic vessels during development, but also occurs in adults during wound healing, inflammation, primary and metastatic tumour growth (Ducoli and Detmar, 2021). Each of these conditions results in a significantly altered microenvironment which results in increased inflammation and fluid accumulation which stimulate lymphatic remodelling (Mazzone and Bergers, 2019; Oliver et al., 2020). Blood vessel growth (angiogenesis), largely grow by formation and extension of filipodia-rich tip cells and lateral inhibition of neighbouring endothelial cells. This generates heterogeneity amongst the endothelium and results in differing phenotypes such a stalk, phalanx and transition endothelial cells (Goveia et al., 2020). Together these form a growing endothelial sprout that will anastomose with nearby sprouts to form a new vessel (Cruys et al., 2016). Although not currently characterised to the same level of resolution, lymphangiogenic vessels also display some degree of tip/stalk selection (Yuan et al., 2002; Benest et al., 2008; Xu et al., 2010) but it should be noted that lymphangiogenic features (including filopodia extension and proliferation) do appear in canonically non-tip cell or sprout like locations (Benest et al., 2008; Baluk et al., 2009). The lumenised neovessel will undergo further phenotypic switches; remodelling of the basement membrane, formation of endothelial cell junctions and a metabolic shift away from glycolysis and initiate state of quiescence (De Bock et al., 2013a; Wilhelm et al., 2016). During endothelial cell quiescence, the balance of activating to destabilising factors is in balance resulting in a stable and functional vasculature (Li et al., 2019).

**FIGURE 2:**
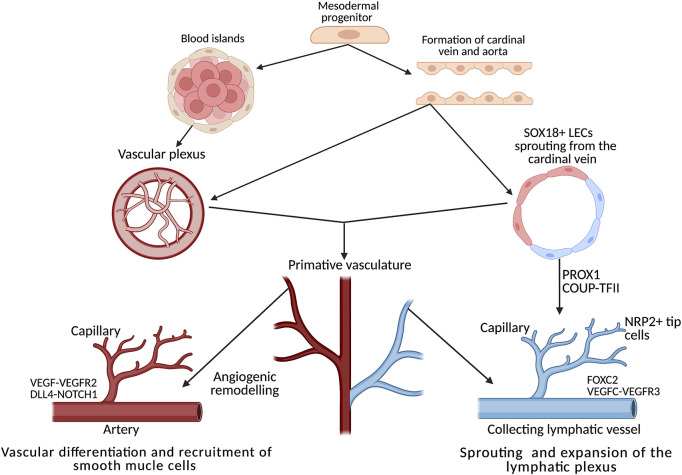
Development, differentiation and separation of the blood and lymphatic networks. Both networks originate from progenitors in the mesodermal layer of the embryo. From the primitive vascular plexus, transcription factors activate the innate genetic programme, resulting in extensive remodelling cascades throughout embryogenesis, forming two distinct networks of vessels. In the heart, the lymphatic plexus is remodelled and guided by tissue-resident macrophages through direct interaction between the lymphatic endothelial cells and the macrophages. Adapted from De Val and Black (2009) and Adams and Alitalo (2007). Created using BioRender.com.

Endothelial phenotypic switching (from quiescence to alternative states) underpins both lymphangiogenesis and angiogenesis; both processes require migration, proliferation and the metabolic remodelling of a quiescent endothelium (Kalucka et al., 2018). The process of angiogenesis, which has drawn more research interest thus far compared to lymphangiogenesis, can be used as a conceptual model, allowing parallels to be drawn between the processes. From the initial blood islands formed of progenitor cells, a plexus of vascular vessels is formed (Figure 2), these progenitors receive frequent remodelling until the primitive embryonic vasculature is recognizable (De Val and Black, 2009). This is not directly analogous to the entire lymphatic system, but local expansion of local lymphatics in mesentery (Benest et al., 2008), dermal tissue (Braverman and Yen, 1974) and cardiac tissue heart might suggest some analogy (Stone and Stainier, 2019). The specific differentiation mechanisms are excellently described here (Stone et al., 2021).

## Endothelial Quiescence as a Physiologically Active, but Not Activated State

Endothelial cells, which line blood vessels (blood endothelial cells) and lymphatic vessels (lymphatic endothelial cells) metabolise more glycolytically than most cells, therefore consuming very little oxygen in a quiescent, stationary state (Wilhelm et al., 2016). Quiescent endothelial cells still require energy to generate new biomass, protect against oxidative stress and are still involved in homeostatic processes (De Bock et al., 2013a). Oxidative phosphorylation in comparison requires more oxygen, generates reactive oxidative species and cannot take place in hypoxic regions. Furthermore, as ECs grow they increase their glycolysis which contributes to their proliferation and survival in hypoxia tissue (De Bock et al., 2013b). In order for migration to take place, the cytoskeleton must be remodelled, an energy-demanding process forcing the switch from a low energy state of quiescence to a more active metabolising, migrating state (De Smet et al., 2009). Transcriptionally, these high-energy demanding cells, display a different transcriptional profile than quiescent endothelial cells (Kalucka et al., 2018). This is also true of the lymphatic endothelium (Wong et al., 2017; Yu et al., 2018).

## Lymphatic Vessels Lose Quiescence as They Grow

In order to establish the lymphatic network, an innate genetic programme is activated by transcription factors in early progenitor cells. Transcription factors are small proteins which act to increase or decrease the expression of genes by binding to promoter sequences of DNA. In establishing the network, LECs must be able to grow and sprout from existing vessels, this requires the cell to be able to respond to growth signals from the environment, such as vascular endothelial growth factor (VEGF)-C which is recognised by vascular endothelial growth factor receptor-2 (VEGFR2) and 3 (VEGFR3) which are both expressed by differentiated LECs (Tammela and Alitalo, 2010; Deng et al., 2015). VEGF-C stimulation can induce translocation of VEGFR2 which results in heterodimers of VEGF-R2 and VEGF-R3 on the cell membrane (Kaipainen et al., 1995; Xu et al., 2010), VEGFR3 can also homodimerize to recognise VEGF-C stimuli (Kukk et al., 1996; Joukov et al., 1997). This activation results in the extracellular signal-regulated kinases (ERK) and protein kinase B (AKT) signalling cascades, which are essential for migration of the lymphatic endothelial cell (Deng et al., 2013, 2015). In adults, lymphatic growth is often pathological lymphangiogenesis, and normally activated in response to injury or disease (Adams and Alitalo, 2007). For example, during inflammation, VEGF-C is produced by macrophages, encouraging the growth of LECs nearby to sprout towards the injury as well as inducing hypertrophy of the collecting lymphatic vessels (Cursiefen et al., 2004; Maruyama et al., 2005). This facilitates the immune response by mobilising dendritic cells and increasing capacity of the vessel to carry lymphatic fluid (Mazzone and Bergers, 2019), and therefore contributes to restoration of tissue homeostasis and resolution of inflammation. When the lymphatic network is established, LECs are quiescent (Sabine et al., 2015); however, upon receiving further cytokine signalling, are stimulated to re-enter the cell cycle (Geng et al., 2020). During a shift to back the quiescent phenotype the non-draining lymphatic sprouts will have generated a lumenised vessel (Geng et al., 2020), resulting in lymph fluid imparting shear stresses upon the lymphatic endothelium. Integration of mechanotransductive signals with transcriptional regulation is a fundamental mechanism for restoring the lymphatic endothelium to a non-activated, and quiescent phenotype.

## Heterogeneity: How Different are Each of the Endothelial Cells Within a Lymphatic Vessel?

As we begin to explore underlying mechanisms responsible for heterogeneity within the cells in the lymphatics, it is critical to note there heterogeneity between different classifications of lymphatic vessel, and terms such as capillaries, collecting and conduit vessels are used to classify the vessels into the “lymphatic tree hierarchy.” Capillaries and collecting vessels significantly differ in morphology and function (Figure 1). The transcriptional expression profile of these different types of vessels could offer early glimpses into understanding quiescent vs. activated phenotypes, as capillaries are the likely site of early phenotyping switching in response to growth stimuli. Any heterogeneity could unveil mechanisms enabling dynamic movement along a spectrum of activated and quiescent phenotypes. Whereas, collecting vessels are mature, established vessels and are likely to be at the quiescent side of the phenotypic spectrum. This is a developing area of interest to many, results from a recent publication by Hernández Vásquez et al. (2021) compared the expression profiles of dermal capillary LECs to collecting vessel LECs in adult mice. This work identified several noteworthy genes of interest, including FOXP2 (discussed later) as a major regulator of collecting vessels morphology, whereas LYVE-1, MAF and CXCL12 were all significantly enriched in lymphatic capillaries, consistent with a more activated/sprouting phenotype (Rondon-Galeano et al., 2020; Hernández Vásquez et al., 2021). Furthermore heterogeneity is apparent between lymphatic vessels from different tissue bed, but less is known about the heterogeneity within a specific vessel bed. Most capillary LECs used in experiments are dermal (commonly isolated from foreskin or breast reduction tissue), however, delving deeper into different organs we see a difference in expression of certain transcription factors, by Wong et al. (2018). Interestingly, intestinal lymphatics known as lacteals, are continuously regenerated throughout adulthood, which aid the lipid absorption in the intestinal villi (Nurmi et al., 2015; Wong et al., 2018). Facilitating this regeneration was high Dll4 expression in these tip cells (Bernier-Latmani et al., 2015). Dll4 is a major driver of blood EC heterogeneity during angiogenesis (Hellstrom et al., 2007; Suchting et al., 2007) and therefore it is probable that similar mechanisms could underpin lymphatic EC heterogeneity too. The presence of valves in lymphatic endothelial cells, made up of specialised lymphatic endothelial cells, within collecting vessels (Figure 1) are also a source of heterogeneity amongst the LECs within the vessel. Single-cell RNAseq (scRNASeq) has enabled researchers to explore cellular heterogeneity at the transcriptomic levels which allows different cellular phenotypes to be identified. Murine lymph nodes were disaggregated and scRNASeq performed (Takeda et al., 2019) upon the LEC populations. A similar approach to explore how quiescence vs activation is manifested in the LEC populations will be an exciting avenue to explore in future work. This is only recently becoming clear from blood endothelial work, but excellent progress has started to explore how collecting lymphatics differ from lymphatic capillaries (Hernández Vásquez et al., 2021) and valve LECs vs. non-valve LECs (Takeda et al., 2019); offering an insight into intralymphatic endothelium heterogeneity. Thus, heterogeneity between inter-vessel LECs and inter-organ must be taken into account before truly understanding intra-vessel LEC heterogeneity.

## Prospero Homebox 1 (PROX1) Interacts With SOX18 and Is Key for Lymphatic Specification

**FIGURE 3:**
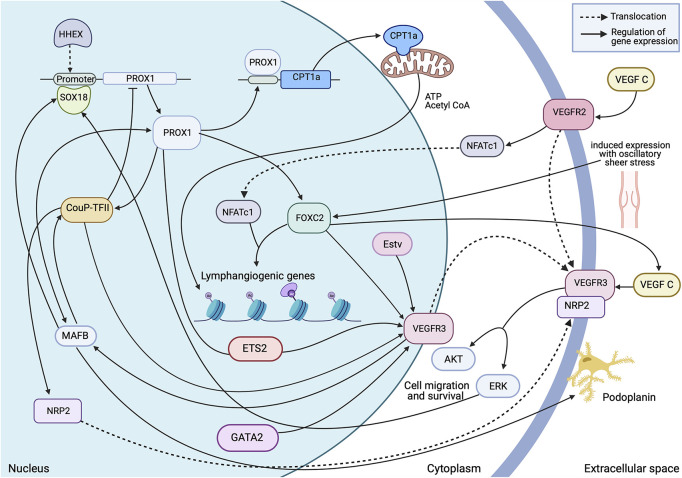
Transcriptional insight into establishment and regulation of the quiescent lymphatic endothelial phenotype. A dynamic and interchangeable network of transcription factors are involved in the complex signalling which differentiates and maintains expression of essential proteins within the cell. This allows the cell to respond accordingly to external stimuli and retain structural stability in areas of high stress. Made with Biorender.com.

The process of lymphatic endothelial cell differentiation, vessel formation and overall maintenance, requires energy, therefore the metabolism of the lymphatic endothelial cell which form the lymphatic endothelium is a key interest in delineating the mechanisms of developmental and pathological lymphangiogenesis. Metabolic activity in the cell is adapted to its phenotype. Endothelial cells can switch between quiescent and proliferating states, and sufficient, differing energy requirements must be met to maintain this state. As the lymph is enriched with nutrients, the LECs must be able to tolerate a high glucose concentration (which is common to all EC, whether blood or lymphatic), and a relatively low oxygen concentration (unlike BECs which are oxygen rich) (Moyon et al., 2001; Schoors et al., 2015; Wong et al., 2017). This results in anaerobic glycolysis as a primary source of ATP (Yu et al., 2018; Jiang et al., 2021), allowing for the generation of energy at sites of filopodia formation. Thus avoiding the need for transportation of ATP from the mitochondria, which are excluded from the thin protrusions (Lee et al., 2018; Li et al., 2019). PROX1 regulated gene expression enhances energy production further by binding the carnitine palmitoyl transferase 1a (CPT1a) promoter, an enzyme which shuttles fatty acids in the mitochondria for oxidation, to increase fatty acid oxidation and acetyl CoA production. Consequently, along with acetylase p300, histones associated with LAG genes are acetylated (Figure 3) this makes the promoters more accessible to PROX1 for transcription (Li et al., 2019).

Through cloning of the PROX1 promoter and confirmation by chromatin immunoprecipitation (ChIP), it was shown that PROX1 is directly activated by SOX18 which binds to a 4 kB fragment of DNA in the PROX1 promoter, through cooperation of SoxA and B sites (François et al., 2008). PROX1 expression is maintained throughout the vasculature, there are elements within this fragment that regulate the expression of PROX1 in the LECs after SOX18 expression has diminished (François et al., 2008). Examining the differentially expressed genes between BECs and LECs, PROX1 emerged as the major regulator of LEC identity. Out of the 300 differentially expressed genes, PROX1 directly regulated 15. In addition, when overexpressed in BECs, PROX1 is able to suppress expression of BEC specific genes such as STAT6 and integrin α5 (Petrova et al., 2002). The exact signal cascades involved in PROX1 induced LEC differentiation are yet to be fully characterised, but by using PROX1 overexpression and knockdown allowed identification of PROX1 effector proteins to be identified. Mishima et al. (2007) found forced PROX1 overexpression in human umbilical vein endothelial cells (HUVECs) and LECs induced a morphological change, in which a sheet formation was inhibited and altered cell morphology was reported (Mishima et al., 2007). A crucial component of this cascade is integrin α9, which is transcriptionally regulated by PROX1, and can reverse the morphological changes induced by PROX1 by blocking its activity. LEC motility was increased with PROX1 overexpression, specifically chemotaxis towards VEGF-C, demonstrating LEC identity, migration and shift towards a more plastic and activated phenotype is masterminded by PROX1 (Mishima et al., 2007). This is further reinforced with the finding that PROX1 contributes to transcription control of CPT1A expression, which in turns shifts lymphangiogenic metabolism away from oxidative phosphorylation (Yoshimatsu et al., 2011) and towards glycolysis and fatty acid metabolism (Wong et al., 2017).

PROX1 is essential for establishing and maintain lymphatic identity, however, over time, its expression is decreased (Ma and Oliver, 2017). Cho et al. (2019) demonstrated YAP and TAZ appear to inhibit PROX1 activity, with YAP/TAZ activity decreased by Hippo pathway signalling. In order for initial LEC budding from the cardinal vein in mouse embryos, this Hippo signalling is increased, lowering YAP/TAZ and allowing establishment of the early lymphatic vasculature (Cho et al., 2019). VEGF-C stimulation of human dermal lymphatic endothelial cells induced localisation of YAP in the cytoplasmic compartment along with an observed increase of VEGFR3 phosphorylation. Consistently, there was increased phosphorylation of LATS1/2 and YAP phosphorylation (key mediators of the Hippo pathway) in addition to YAP phosphorylation increases eventually leading to decreased YAP target genes and PROX1 expression. Pinpointing the role of VEGF-C signalling on influencing the expression of lymphangiogenesis dependent transcription factors (Cha et al., 2020).

SOX18 [SRY (Sex Determining Region Y) box 18] is a member of the SRY-related high mobility group domain family of developmental transcription factors. SOX18 is the first lymphatic marker to be expressed during mouse embryogenesis, prior to PROX1 (François et al., 2008). Detected as early as 9 days post conception, SOX18 positive cells were reported in the cardinal vein, and when at later stages the same population of cells expressed PROX1 and CD31, François et al. (2008) suggested these cells were precursors to the lymphatic vasculature. SOX18 expression is not maintained throughout development, as by 14 days post conception expression had subsided, suggesting that SOX18 acts as a molecular switch to activate differentiation of the endothelial cells to a lymphatic phenotype (François et al., 2008). This switch is induced by ERK signalling, which in turn is regulated by RAF1, a mitogen activated 3 kinase (Deng et al., 2013). Interestingly a study by Deng et al. (2013) which showed excessive RAF1 activation induced uncontrolled blood to lymphatic vessel phenotype (lymphangiectasia), revealing a crucial role of ERK signalling in this early developmental stage (Deng et al., 2013). Vascular cell adhesion molecule 1 (VCAM1) shares a spatiotemporal pattern of expression as SOX18, sparking a suggestion of crosstalk within the pathways controlling the LEC phenotype. VCAM1 is expressed on the surface of activated endothelia, Hosking et al. (2004) discovered three SOX18 binding sites in the VCAM1 gene, and specifically the SoxB site that is essential for transactivation of VCAM1 expression (Hosking et al., 2004) suggesting that careful control of SOX18 expression determines aspects of lymphatic quiescence as VCAM1 is a key mediator of an activated phenotype.

## Chicken Ovalbumin Promoter Transcription Factor II (CouP-TFII) Interacts With PROX1 During Lymphatic Proliferation

CouP-TFII is an orphan member of the steroid/thyroid hormone superfamily. Expression of CouP-TFII has been reported by Yamazaki et al. (2009) to be essential in segregating lymphatic vasculature from the primitive veins. A physical interaction between PROX1 and Coup-TFII was discovered by ChIP this was found to centre around the cyclin E1 promoter, an important molecule involved in S phase of the cell cycle (Petrova et al., 2002; Yamazaki et al., 2009), excess CouP-TFII was found to inhibit the proliferation inducing ability of PROX1. CouP-TFII also acts independently of PROX1, required after the initial sac formation to maintain the lymphatic identity. As LECs are identified by common marker expression, altered expression can be used as evidence for a change in behaviour of the cell. For example, Lin et al. showed that a CouP-TFII endothelial specific deletion caused a decreased in expression of the classic lymphatic markers such as LYVE1, PROX1, NRP2 or VEGFR3. VEGFR3 and NRP2 are both key regulators of lymphatic quiescence and modify VEGF-C signalling in the lymphatic EC (Yuan et al., 2002; Xu et al., 2010; Bouvrée et al., 2012); which is the major lymphangiogenic signalling pathway. Interestingly, these ECs ectopically expressed more commonly known BEC markers instead, suggesting CouP-TFII is involved in maintaining the identity of LECs in early vessel formation, prior to full maturation. Specifically, CouP-TFII is a positive regulator of Neuropillin-2 (NRP2) expression, acting through the SP-1 binding site located in the promotor (NGFIA) of NRP2 (Lin et al., 2010). NRP2 has previously been identified as a co-receptor for VEGFR3 (Yuan et al., 2002). Disruption of NRP2 selectively disturbs sprouting of LECs in response to VEGF-C, suggesting NRP2 drives the tip cell phenotype, as stalk cell morphology was unchanged. Tip cells lead new sprouting vessels; thus, a deficiency of tip cells results in less growth of the lymphatic network (Xu et al., 2010).

## GATA2 Regulates VEGFR3 Expression and Contributes to Lymphatic Remodelling

GATA2 is a member of the zinc finger transcription factor family. Work by Frye et al. (2018) demonstrated that GATA2 was upregulated in migrating LECs from the Cardinal Vein (CV), analysis revealed that the change in matrix stiffness, as the endothelial cells migrate into the surrounding parenchyma activates GATA2 expression. From the CV, LECs begin to form primitive vessels which make up vessel beds (10.5 days post conception), beyond this, the increase in interstitial fluid pressure results in a stretch response, resulting in enrichment of genes involved in cell matrix adhesion, junctional organisation, migration and vascular development. A specific gene of interest is VEGFR3, which acts as a receptor for VEGF-C, GATA2 binds directly to intron 1 of VEGFR3 to regulate its expression. This is important as VEGF-C is crucial for sprouting and migration of LECs. Loss of GATA2 substantially downregulated VEGFR3, and LECs failed to respond to VEGF-C. In normal development, the stretch-activated phosphorylation of VEGFR3 initiates a signalling cascade which activates proliferation and vessel growth. The interstitial flow within the premature vessels is crucial in inducing mechanical forces which further shape the vasculature, for example, through inhibition of Neurogenic Locus Notch homologue protein 1 (NOTCH1) sprouting is promoted and Krüeppel-Like Factor 2/4 (KLF2/4) induces proliferation (Frye et al., 2018). By day 15 post conception, the flow at branch points induces a GATA2 and FOXC2-dependent quiescence, as LECs are correctly targetted to important points where the LV and BV connect (Frye et al., 2018). Matrix metalloproteinase signalling was also increased by the change in matrix stiffness as a result of migration, this is crucial for lymphangiogenesis as the surrounding extracellular matrix must be remodelled to facilitate sprouting of the vessels (Detry et al., 2012; Frye et al., 2018).

Collecting lymphatics are distinguishable from capillaries by size, coverage by smooth muscle cells and pericytes, and the presence of valves (Figure 1). These valves are not only present inside the lymphatic vessels but also crucially at the junctions between the lymphatic vessel and the blood vessel. The valves are formed by intercalations of LECs with a type of vascular endothelial cell which is PROX1 and also PCAM positive (Sathish Srinivasan and Oliver, 2011). Lympho-venous valves require PROX1 and Coup TF-II complex formation to regulate the dosage of PROX1, deletion of even one copy of PROX1 is enough to induce abnormal connections between the two systems, as differentiation into valve cells is compromised (Sathish Srinivasan and Oliver, 2011). GATA2 has also been implicated in valve morphogenesis, as a mechanosensory transcription factor, it recognises the oscillatory shear stress at vessel branch points. By using GATA2 deletions, we can infer importance as the resulting embryos lacked these valves and presented with blood inside the lymphatic vessel, which is a characteristic of improper formation of valves at the lymphovascular junctions (Frye et al., 2018). GATA2 mutations are responsible for Emberger syndrome, carriers of this mutation are predisposed to leukaemia and lymphoedema, this is due to the crucial role of GATA2 in the differentiation of LECs specifically in the lymphovenous vales (Geng et al., 2016).

## FOXC2 Plays a Key Role in Lymphatic Maturation

PROX1 associates with regulatory elements of Forkhead box C2 (FOXC2) (Cha et al., 2016). FOXC2 has roles in angiogenesis, is essential in lymphatic vasculature and is a known marker of the lymphatic valve (Kume, 2008; Norrmén et al., 2009; Takeda et al., 2019; Xiang et al., 2020). In mice, FOXC2 is expressed at 8.5 days post conception in the normal developing heart, blood vessels and limbs, expression in the endothelial cells is recorded at between 9.5 and 10.5 days post conception, along with PROX1 and LYVE1 (Dagenais et al., 2004). This subset of endothelial cells are involved in migration and sprouting to form immature mesh-like networks of vessels, which are organised in a cranial to caudal manner, these separate networks fuse creating major lymphatic pathways (Dagenais et al., 2004; Norrmén et al., 2009). Part of this process includes a dramatic remodelling of the mesenteric plexus, this is where the differentiation of collecting vessels and capillaries becomes apparent. FOXC2 expression is thought to be induced by oscillatory sheer stress (Figure 3), as the highest FOXC2 levels were found in endothelial cells which form part of the valves, which are exposed to the most disturbed flow (Sabine et al., 2015). Oscillatory sheer stress has significant influence of the gene expression patterns of LECs, controlling over 800 genes, however, when receiving a FOXC2 inducible knock out, these cells responded abnormally to shear stress. Oscillatory sheer stress normally induces growth arrest to protect the vessel structure by decreasing cell proliferation. However, knockdown FOXC2 *in vitro* generated a TAZ dependent proliferation and increased cell death, suggesting FOXC2 has an important role in maintaining quiescence in areas of high shear stress (Sabine et al., 2015). As maturation progresses, FOXC2 expression decreases in the areas not underconstruction, such as the intraluminal segments between valves (17.5 days post conception) (Norrmén et al., 2009). Even at maturity, high FOXC2 expression is maintained in the valves, suggesting valve LECs are molecularly distinct from neighbouring cells in the trunk of the collecting vessel (Norrmén et al., 2009). It would be interesting to compare the LECs in the collecting lymphatic vessels which are quiescent, to those in the capillaries which are likely heterogenous as they are reactive to lymphangiogenic stimuli. There is also growing evidence that modulating this heterogeneity is the level of VEGFR3 expression (Zhang et al., 2018).

It is suggested that FOXC2 could cooperate with VEGFR3 to specify the phenotype of the lymphatic vessel, as FOXC2 is expressed in valves, required in the larger collecting vessels, compared to smaller capillaries which lack valves, smooth muscle coverage and full coverage by a basal lamina (Alitalo et al., 2005; Oliver and Srinivasan, 2008). VEGFR3 is not downregulated in FOXC2-/- mice, but in VEGFR3-/- embryos, mRNA for FOXC2 is decreased, confirming that VEGFR3 is upstream of FOXC2 and may have a role in its expression (Petrova et al., 2004). FOXC2 regulates the expression of VEGF-C (Oliver and Srinivasan, 2008) which has been established as an essential chemotrophic factor and an activating ligand for VEGFR3, which will permit an autocine loop regulating the LECs own quiescence.

The nuclear factor of activated T cells (NFATc1 specifically) is a calcium-sensitive transcription factor, which is also involved, sharing expression patterns with FOXC2 and regulation by PROX1 (Norrmén et al., 2009). NFATc1 needs to localise into the nucleus where it interacts with other nuclear and transcription factors (such as AP1, nuclear factor κB, Foxp3, GATA) to form complexes on DNA. VEGF-C acting on VEGFR2 induces translocation of NFATc1 to the nucleus, this receptor is expressed collecting lymphatics and valves, and is thought to promote an increase in vessel size. The importance of this transcription factor is elucidated with experimental deletion of NFAT signalling, whereby lymphatic remodelling and maturation is defective, sharing a similar phenotype to FOXC2-/- mice. The expression of lymphatic capillary markers, lack of valves and impaired sprouting seen in these NFAT-/- mice summarises to a hyperplastic phenotype, which is further exacerbated by loss of a FOXC2 allele. Investigating the link between FOXC2 and NFATc1 further, Norrmén et al. (2009) established that the genes are expressed independently but are found to co-regulate transcription of downstream genes, as ChIP analysis of primary LECs revealed NFAT-binding sites in close proximity of FOXC2 sites (Norrmén et al., 2009). Thus, both NFATc1 and FOXC2 share a role in establishing the collecting lymphatic phenotype.

## Shear Stress as a Primary Determinant of Quiescence?

Elucidating the FOXC2/NFATc1 pathway further, a newly discovered downstream target, FOXP2, previously implicated in speech development in humans (Co et al., 2020), has been identified by Hernández Vásquez et al. (2021) as another marker of collecting lymphatics in both mouse and human models (Hernández Vásquez et al., 2021). ChIP sequencing had previously linked FOXC2 and FOXP to roles in the lymphatic system (Norrmén et al., 2009), but it only recently this role has been investigated further. The expression of the transcription factor FOXP2 was induced by oscillatory shear stress, acting downstream of FOXC2 to help regulate the collecting lymphatic phenotype and valve development. The role of oscillatory shear stress is appearing to be a key determinant in examining transcriptional regulation in establishing a functional lymphatic network. But it is not just disturbed, oscillatory shear stress that influences the vessel transcriptional profile, regular laminar stress present in non-sprouting, mature vessels, acts to maintain quiescence in these cells. LECs at the growing front of sprouting vessels grow *via* projecting extensions of the cell membrane, these projections are not lumenised, so are not exposed to the circulating lymph, or the fluid dynamics that go with it. This allows tip cells expressing markers such as DLL4 to enhance VEGF-C signalling and allowing lymphatic growth. Meanwhile, stalk cells in these vessels do not express DLL4, Geng et al. (2020) found sphingosine 1-phosphate receptor 1 (S1PR1), a G-protein coupled receptor, antagonises the VEGF-C signalling, enhanced by laminar shear stress independent of S1PR1, allowing the stalk cells to maintain their quiescence. S1PR1 is thought to act by activating Claudin 5, a tight junctional protein, contributing to proper cell-junction formation in mature lymphatic vessels (Geng et al., 2020). Therefore, consolidating the interplay between how shear stress regulates differential transcription factor activity and therefore how this contributes LEC phenotype is one of great promise. Of note, it is well recognised that LEC, *in vivo*, are able to grow along “fluid” channels *in vivo* during tissue regeneration (Boardman and Swartz, 2003). The transcription factor cascades underpinning such events are unknown but would likely reveal novel aspects consolidating the activated migratory and quiescence switching in response to altered fluid dynamics.

Lymphatic endothelial cells are very sensitive to changes in lymphatic flow, functioning in a narrow window of exposure to shear stress (Baeyens et al., 2015). Many pathological conditions such as chronic heart disease (Boehme et al., 2021) and lymphodema (Scallan et al., 2016), result in chronically elevated lymphatic flow, which can overstimulates the signalling pathways in place to protect the lymphatic endothelium. As described above, through FOXC2, induces a growth arrest allowing maturation of vessels repressing the expression of cell-cycle progression genes (Sabine et al., 2015). This allows the cell to adapt to the high stress conditions, limiting damage as cell-cell contacts are reinforced and motility is limited (Sabine et al., 2015). Over time, if this high flow is maintained, the cells face constant interstitial pressure induced-stretch, β1 integrins on the surface of LECs translate this stretch to VEGFR3 tyrosine phosphorylation which results in signalling for LEC proliferation (Planas-Paz and Lammert, 2013).

KLF-2 is another mechanosensitive transcription factor, the expression of which is upregulated in both oscillatory and laminar flow (Choi et al., 2017). KLF2 is responsible for the flow-induced expression of VEGF-C (Choi et al., 2017) and disruption of PPAR-γ signalling (Morris et al., 2018). PPARγ is a part of the nuclear hormone receptor superfamily. In LECs, PPAR-γ expression is decreased in shear stress conditions. In low stress conditions, PPARγ signalling inhibits expression of NADPH oxidase, increasing bioavailable nitric oxide, an important regulator of vascular tone. In LECs exposed to chronic shear stress have increased NADPH expression, increased ROS -which further scavenge bioavailable NO- disrupting NO homeostasis, this dysfunction is restored with KLF2 knockdown (Morris et al., 2018), isolating responsibility of this transcription factor in this signalling in shear stress conditions.

Hypoxia inducing factor-1α (HIF-1α) is a transcription factor commonly associated with inflammatory states and the hypoxic response regulates over 1,000 target genes (Semenza, 2013). HIF-1α has been associated with lymphangiogenesis in malignancy (Schoppmann et al., 2006; Liang et al., 2008) but was discovered by Boehme et al. (2021) to have a critical role in turnover of LECs which are chronically exposed to high stress conditions (Boehme et al., 2021). In relation to a pathological model of coronary heart disease, in which there is a chronic increase in pulmonary lymphatic flow, the LECs in the high stress conditions increased HIF-1α expression despite not experiencing hypoxic conditions (Boehme et al., 2021). This suggests HIF-1α may be regulated by mechanotransductive forces on the lymphatic endothelium, specifically the ROS from mitochondria experiencing stress, which are central upstream mediators of HIF-1α. This suggests an interface mechanotransductive signals and quiescence through HIF-1α involvement (Boehme et al., 2021).

## MAFB Contributes to Branching Lymphatic Morphogenesis

Recent work by Dieterich et al. (2020) revealed a role of lymphatic V maf musculoaponeurotic fibrosarcoma oncogene homolog (MAFB) in transcriptional regulation of vascular patterning. In LECs, this expression upregulated VEGF-C/R3 signalling *via* direct binding to MAF recognition elements (MARE) in the promoter and enhancer in the DNA sequence (Dieterich et al., 2020). Transcriptomic analysis indicated MAFB is involved in the early induction of SOX18 expression, thus impacting PROX1 production through this signalling pathway (Dieterich et al., 2015). This work was followed up by Rondon-Galeano et al. (2020), using a CRIPSR/Cas9 mouse model, these mice had a perinatal death associated with cyanosis. Upon investigation, dermal lymphatics in these mice had mild and transient delay in development. However, in the diaphragm, MAFB was necessary for patterning the lymphatics that developed in the mutant mice were broader and covered a larger area of the diaphragm (Rondon-Galeano et al., 2020). Other elements of the signalling cascade linked to MAFB include PROX1, LYVE1 and podoplanin. Global knockout of MAFB induces an hyperbranched phenotype in the developing LV, with decreased overall growth, suggesting MAFB is involved in refining the branching of the LV capillaries, as depletion increased the number of junctions and cord segments (Dieterich et al., 2020). Podoplanin activates platelet aggregation, this separates the primary lymph sac from the CV. Specifically, podoplanin activates c-type lectin receptor 2, which acts on SLP76 to activate syk (a tyrosine kinase) in platelets (Tammela and Alitalo, 2010). Podoplanin is expressed on the membrane of LECs as are glomerular podocyte, promoting adhesion, migration and tube formation. Pups with podoplanin KO die at birth from respiratory failure, and displayed defects in lymph patterning and function (Oliver and Srinivasan, 2008). There was an absence in formation of a fluid functional network of lymphatics in these mice, as deeper lymphatics fail to form connections with capillaries at the surface, thus showing defects in migration of LECs and of lumen formation (Oliver and Srinivasan, 2008).

## ETS-Domain Transcription Factors

ETS-domain transcription factors are a family of 19 endothelially expressed transcription factors characterized by highly conserved DNA binding domain and the DNA-binding consensus sequence GGA(A/T) (Hollenhorst et al., 2007). Interestingly, ETS2 and Etv2 were found to be expressed in BECs as well as LECs (Dejana et al., 2007; Yoshimatsu et al., 2011; Davis et al., 2018). Yoshimatsu et al. (2011) identified the expression of ETS2 and its co-localization with PROX1 in nuclei of LECs. Further analysis revealed that ETS2 physically and functionally interact with PROX1. In addition, their work highlights the synergistic enhancement of Ets2 and PROX1 in expression of VEGFR3. Consistent with the effects on expression profile of VEGFR3, ETS2 induces LEC migration towards VEGF-C (Yoshimatsu et al., 2011). In the light of the previous data ETS2 is reported as a pivotal pro-lymphangiogenic factor in collaboration with PROX1 during lymphangiogenesis (Yoshimatsu et al., 2011). Furthermore, another transcription factor of interest, Etv2/Etsrp, has been investigated as a lymphangiogenic initiator directly promoting the expression of VEGFR3 within the posterior cardinal vein (Davis et al., 2018). Using *in vitro* differentiated mouse embryonic stem cells, Etv2 ChIP-Seq analysis revealed specific Etv2 binding peaks present within VEGFR3 and LYVE1 promoter/enhancer regions (Liu et al., 2015). The VEGFR3 promoter is a likely direct target of Etv2, containing an evolutionarily conserved FOX:ETS domain that is bound by Etv2 and FOXC2 transcription factors (De Val et al., 2008). Further analysis using luciferase reporter studies in zebrafish embryos and ECs suggested Etv2 activates both VEGFR3 and LYVE1 through direct binding to their promoter/enhancer regions, and that the function of these enhancers is conserved among different vertebrates (Davis et al., 2018).

Of the transcription factors regulating endothelial cell physiology, haematopoietically expressed homeobox (HHEX), is composed of a proline-rich domain and a highly conserved homeodomain (Ho et al., 1999). Intriguingly, HHEX was found to be expressed by endothelial cells in both blood and lymphatic vessels from the earliest step of sprouting angiogenesis and lymphangiogenesis from the PCV until adulthood. Further ChIP analysis in blood endothelial cells have revealed putative HHEX binding sites upstream of the PROX1 transcriptional start site. On contrary, HHEX lacks direct binding to enhancer regions of VEGF-C/VEGFR3. Collectively these data support a model where HHEX is an upstream transcriptional regulator of VEGFR3/VEGF-C/PROX1, acting directly to PROX1 transcriptional site (Gauvrit et al., 2018; Figure 3).

## Lessons From Single Cell Sequencing

The transcriptomic exploration of lymphatic vasculature has been greatly expanded through the use of single-cell RNA-Seq (scRNA-seq), probing gene-expression data at the resolution of single-cells (Chen et al., 2019; Xiang et al., 2020). Whilst studies had previously suggested heterogeneity among LECs (Park et al., 2014; Ulvmar et al., 2014; Iftakhar-E-Khuda et al., 2016), single-cell techniques have allowed further characterisation of LEC heterogeneity with six transcriptionally distinct PROX1^+^ LEC clusters (clusters I-VI) being identified in human lymph nodes. Although a highly specialised lymphatic vasculature, such high-resolution analysis has allowed the difference quiescence states to be identified. For example, expression of cell-cell junction, ECM interacting proteins and inflammatory marker expression demonstrate heterogeneity. Following on from the single cell RNA-Seq analysis of human lymph node LECs, the group investigated murine lymph node LN LEC heterogeneity (up to seven specific identities) and compared the findings with the human results (Xiang et al., 2020). Five mouse LEC clusters were identified as shared between mouse and human. The transcription factor FOCX2, showed high expression levels in cells identified as valve cells and is a shared marker gene between mice and humans (Xiang et al., 2020). Another shared marker gene of lymphatic valve cell was the transcription factor GATA2 (Xiang et al., 2020) which has been previously shown to be critical for the development and maintenance of lymphatic valves (Kazenwadel et al., 2015; Riaj Mahamud et al., 2019). Interestingly, the corresponding human LEC cluster to murine valve subset (LEC V) also shows a high expression level of FOXC2 and GATA2 but were detected in a small percentage of cells in the subset. Other transcription factors identified as shared between mouse and human LECs include with heterogenous expression across the lymph node include KLF4 (Takeda et al., 2019; Xiang et al., 2020), which has been demonstrated to be a key regulator of the components of flow-induced LEC proliferation (Choi et al., 2017) and RELB (Takeda et al., 2019; Xiang et al., 2020), a member of the nuclear factor-κB (NF-κB) family (Yang et al., 2019) known to play a key role in the development and function of lymphatic vessels mediated by LECs (Liang et al., 2019).

## Future Perspective and Conclusion

Understanding the lymphatic endothelium will be as important as the blood endothelium as the site of major disease in the coming years. For this to become realised, it will be essential for researchers to understand the transcriptional landscape of the lymphatic endothelium, especially in light of how the transcriptome is dynamic to its microenvironment. This is especially important as sex differences between male and female lymphatic systems are being increasingly recognised as being significant to disease progression in cardiovascular disease (Trincot and Caron, 2019), however, the transcription factor heterogeneity to this of this is not yet clear. Currently, more research is actively questioning how the lymphatic EC is phenotypically different to a blood EC, rather than exploring the heterogeneity within the lymphatic endothelium. As we begin to understand the molecular regulators of lymphangiogenesis, and how lymphatic function is controlled, we will begin to identify how “quiescence to activation” paradigms exist within the lymphatic endothelium, and it will be of future work to establish the significance of this in terms of biology but also the application to disease conditions. This review has focussed on the major transcription factors that are active during lymphangiogenic remodelling, and its relation to a quiescent and mature phenotype. It is hoped that as the identity of further transcription factors are identified, through the use of cutting-edge techniques (such as scRNASeq and advanced ChIP, proteomic and RIME) further mechanistic studies will be able to contribute to our understanding of lymphatic quiescence.

## Author Contributions

ZT, NA, JH, SS, and AB drafted the article. All authors contributed to the article and approved the submitted version.

## Conflict of Interest

The authors declare that the research was conducted in the absence of any commercial or financial relationships that could be construed as a potential conflict of interest.

## Publisher’s Note

All claims expressed in this article are solely those of the authors and do not necessarily represent those of their affiliated organizations, or those of the publisher, the editors and the reviewers. Any product that may be evaluated in this article, or claim that may be made by its manufacturer, is not guaranteed or endorsed by the publisher.
